# Essential oils and their components are a class of antifungals with potent vapour-phase-mediated anti*-Candida* activity

**DOI:** 10.1038/s41598-018-22395-6

**Published:** 2018-03-02

**Authors:** Adam F. Feyaerts, Lotte Mathé, Walter Luyten, Stijn De Graeve, Katrien Van Dyck, Lize Broekx, Patrick Van Dijck

**Affiliations:** 1 VIB Center for Microbiology, KU Leuven, 3001 Leuven, Belgium; 2 Laboratory of Molecular Cell Biology, KU Leuven, 3001 Leuven, Belgium; 3 Department of Biology, KU Leuven, 3000 Leuven, Belgium

## Abstract

Multi-resistant microorganisms continue to challenge medicine and fuel the search for new antimicrobials. Here we show that essential oils and their components are a promising class of antifungals that can have specific anti-*Candida* activity via their vapour-phase. We quantify the vapour-phase-mediated antimicrobial activity (VMAA) of 175 essential oils and 37 essential oil components, representing more than a 1,000 unique molecules, against *C*. *albicans* and *C*. *glabrata* in a novel vapour-phase-mediated susceptibility assay. Approximately half of the tested essential oils and their components show growth-inhibitory VMAA. Moreover, an average greater activity was observed against the intrinsically more resistant *C*. *glabrata*, with essential oil component citronellal having a highly significant differential VMAA. In contrast, representatives of each class of antifungals currently used in clinical practice showed no VMAA. The vapour-phase-mediated susceptibility assay presented here thus allows for the simple detection of VMAA and can advance the search for novel (applications of existing) antimicrobials. This study represents the first comprehensive characterisation of essential oils and their components as a unique class of antifungals with antimicrobial properties that differentiate them from existing antifungal classes.

## Introduction

The worldwide incidence of infectious diseases continues to mount, and causative microorganisms are increasingly showing (multi-)drug resistance. Therefore, the discovery of new antimicrobials and the repurposing of old ones is critical^1–4^. Fungal infections in humans are mostly caused by *Candida albicans* and *C*. *glabrata*, which are both dominant members of the human mycobiome that are genetically and phenotypically very different^5,6^. While *C*. *albicans* is most frequently identified as the causative species, the incidence of infections caused by *C*. *glabrata* is rising, and this species shows an overall lower susceptibility to common antifungals^7,8^.

Many antimicrobials, such as the polyenes, macrolides, echinocandins, penicillins and essential oils (EOs), are natural products or derivatives thereof^9–13^. EOs are complex mixtures of secondary plant metabolites that have a relatively high vapour pressure, are poorly water-soluble and known for exerting a multitude of biological effects, including anti-*Candida* activity^14,15^. EOs contain many EO components (EOCs), which are mostly derived from intermediates of the mevalonate, methylerythritol phosphate and shikimic acid metabolic pathways^15,16^. For determining the composition of the volatile EO fraction analytical methods such as gas chromatography combined with flame ionisation detection and/or mass spectrometry can be used^17^ and for analysis of the non-volatiles EO fraction liquid chromatography would be recommended. Typically, within one EO, a few EOCs are present at high concentrations while the others are present in small or trace amounts^14^.

To quantify the antimicrobial activity of a molecule against a specific microorganism, the minimal inhibitory concentration (MIC) is typically determined, preferably using standardised protocols such as the broth microdilution assay^18–21^. However, standardised protocols are not necessarily suitable for antimicrobials with a high vapour-pressure^22,23^. These molecules may exhibit an antimicrobial activity at a distance that is mediated by their vapour-phase, which permits easy administration by inhalation, treatment of *e*.*g*. porous substances and rapid removal by airing. The vapour-phase-mediated antimicrobial activity (VMAA) is often overlooked, undervalued or neglected. Therefore, we introduce the vapour-phase-mediated susceptibility (VMS) assay which allows a semi-quantitative study of the VMAA. The procedure is based on the Clinical and Laboratory Standards Institute (CLSI) protocol for the broth microdilution assay using standard 96-well plates^24,25^. As such, the VMS assay resembles the previously introduced vapour-phase-mediated patch assay which can be used for the detection of VMAA^23^. Both assays belong to a new class of broth microdilution based assays where a volatile is evaluated for its biological activity in a liquid culture, following its initial evaporation and migration via the vapour-phase.

Here, we quantified the VMAA of a large collection of commercially available EO(C)s against *C*. *albicans* and *C*. *glabrata*. This allowed us to (i) identify EO(C)s with a strong VMAA against pathogenic *Candida* species, (ii) compare the average susceptibility of both yeasts to our EO(C) collection, and (iii) identify EO(C)s showing a differential activity against both *Candida* species. As such, this proof-of-principle study demonstrates that gauging the VMAA of bioactives using the VMS assay can lead to the discovery of novel (applications of) antimicrobials. Hence from a drug discovery perspective, volatiles with VMAA are possibly interesting compounds for the treatment of (*Candida*) infections related to the digestive, vaginal and respiratory tract^26–28^.

## Results

### The VMAA of a volatile spreads symmetrically across a microtiter plate

**Figure 1:**
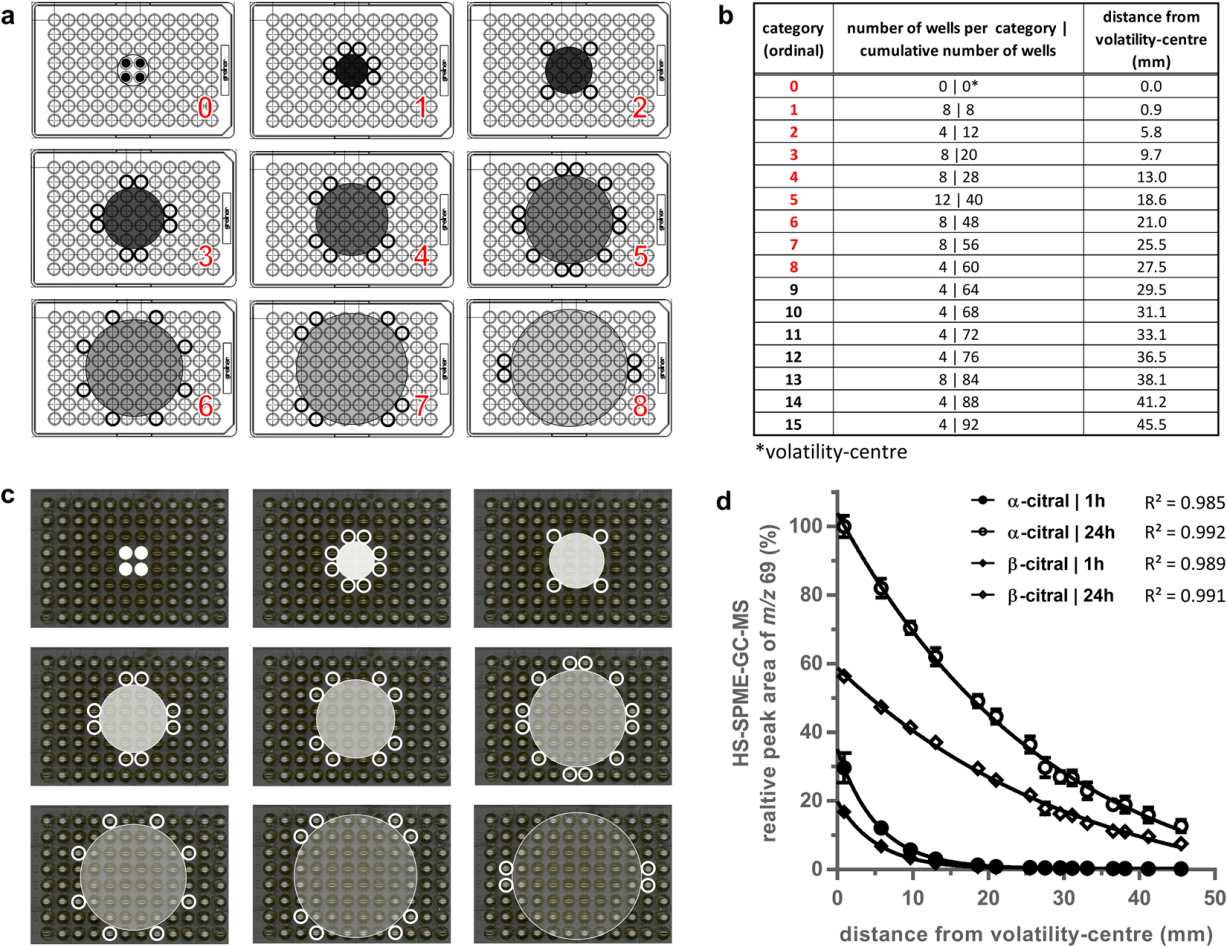
The VMAA of a volatile spreads symmetrically across a microtiter plate. (**a**) Illustrations of the spreading of a volatile in the VMS assay under ideal circumstances starting from the centre four wells (=volatility-centre which corresponds to category 0: upper-left); upper-middle to bottom-right: The first eight categories in which equidistant wells are affected (categories are indicated in red). (**b**) The number of equidistant wells and cumulative number of wells in successive categories with their distance to the volatility-centre. (**c**) Optical scans of the bottom of a 96-well microtiter plate illustrating the VMAA of *Litsea citrata* against *C*. *albicans* after 24 hours of incubation. Panels correspond to categories shown in Fig. 1a. (**d**) Graph illustrating the negative exponential distribution of both enantiomers of EOC citral over the microtiter plate in the VMS assay after one and 24 hours of incubation. Adjusted R² values represent goodness of fit. Each dot represents the relative peak area (extracted ion channel *m/z* 69) in HS-SPME-GC-MS analysis representing the concentration of pooled equidistant wells belonging to the same distance category as a function of their distance to the volatility-centre. Data from three independent experiments are shown and error bars represent standard deviation. The largest average absolute peak area over the different experiments was set as 100%, i.e. α-citral (24 h) at 0.9 mm.

To quantify the vapour-phase-mediated activity in a straightforward manner, we developed the VMS assay and characterised the behaviour of volatiles in this assay using 96-well plates. We hypothesised that, under ideal conditions, a volatile added to the central four wells would spread symmetrically in a spherical manner, thus establishing a concentration gradient across the microtiter plate. A circle enclosing the four wells to which the volatile is added, was designated the volatility-centre (Fig. 1a upper left). Around this centre, concentric circles can be drawn that successively touch the nearest equidistant wells, with each set of wells making up a new distance category. These categories were defined to correct for the different number of wells in different categories and were ranked ordinally, with category one located closest to the volatility-centre (Fig. 1a,b). The distance between the circles and the volatility-centre is the minimum distance that a volatile needs to travel to possibly exert effects in the corresponding wells. Therefore, the content of all wells belonging to one category would be affected equally, due to radial symmetry and can be considered as technical repeats.

Above theoretical model was illustrated by the growth inhibitory VMAA of the EO of *Litsea citrata*, rich in citral (71.80%; SI 1), on *C*. *albicans* in a VMS assay (Fig. 1c). As predicted, growth inhibition clearly increased the closer wells were located to the volatility-centre, and this visual impression was confirmed spectrophotometrically (SI 2). Headspace solid‐phase micro-extraction gas chromatography mass spectrometry (HS-SPME-GC-MS) on the content of the wells of the microtiter plates confirmed the presence of α-citral and β-citral in all wells, as quickly as one hour after addition of the EOC citral (≥95%) and this amount increased over the next hours (Fig. 1d). The largest inhibitory effects were observed in the wells located close to the volatility-centre (Fig. 1c), and they were associated with the highest concentration of citral measured (Fig. 1d).

We defined the inhibitory VMAA (iVMAA) as the categorised cumulative number of wells (Fig. 1a,b), determined by visual assessment and excluding the volatility-centre, in which growth was completely inhibited. When growth was only inhibited in some wells of one category, due to asymmetrical iVMAA patterns resulting from *e*.*g*. uncontrolled airflows, we assigned the intermediate value of 0.5. Only one intermediate categorical value was introduced between every two categories because the different categories can contain a different number of equidistant wells. Therefore, proportionally assigning the fraction of wells affected would result in an unequal number of intermediate values between every two categories.

A spectrophotometric determination of iVMAA termed iVMAA_90_ and defined as the inhibitory VMAA resulting in a 90% reduction of growth as compared to control growth, showed a very high correlation (ρ = 0.991, p < 0.0001; **SI 3**) with iVMAA. This reflects a very high similarity between visual and spectrophotometric assessment and shows that both read-outs are equally valid.

### The MIC of EO(C)s cannot be used to predict their iVMAA and vice versa

**Figure 2:**
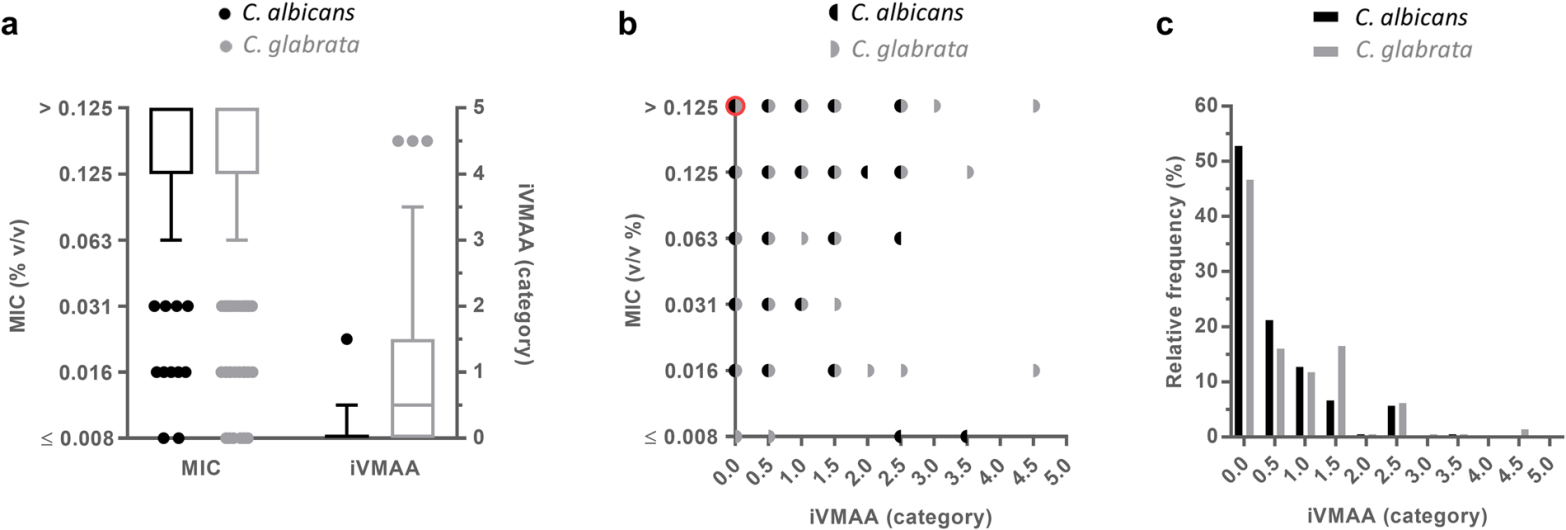
MIC of EO(C)s cannot be used to predict their iVMAA and vice versa. (**a**) Tukey boxplots representing MIC and iVMAA of EO(C) (n = 212) against *C*. *albicans* and *C*. *glabrata*. (**b**) Scatterplot of the correlations between MIC and iVMAA of EO(C)s (n = 212) against *C*. *albicans* (ρ = −0.0376, p = 0.59) and *C*. *glabrata* (ρ = −0.0555, p = 0.42). Red symbol indicates negative control DMSO. (**c**) Histogram with the relative frequency distribution of iVMAA of EO(C)s (n = 212) against *C*. *albicans* and *C*. *glabrata*. The relative frequency was calculated by dividing the number of EOC(s) in each category by the total number of EO(C)s. MIC = minimal inhibitory concentration; iVMAA = inhibitory vapour-phase-mediated antimicrobial activity.

To characterise the vapour-phase-mediated growth inhibitory activity of EO(C)s, we determined the iVMAA of 175 chemically defined EOs (SI 1), and 37 of the most common EOCs (SI 4) against *C*. *albicans* and *C*. *glabrata* (Fig. 2a). As the VMS assay set-up is based on CLSI guidelines for the broth microdilution assay^24,25^, we also determined the MIC of our EO(C) collection against both *Candida* species (Fig. 2a). Despite the highly comparable experimental procedures used to determine the iVMAA and MIC, a comparison between these values for both species showed that the iVMAA of an EO(C) cannot be predicted from its MIC value and vice versa (Fig. 2b**)**.

Nine of the EOCs tested (24.3%) showed an iVMAA larger than 0.5 against both *Candida* species. The greatest activity was observed for EOC citronellal, followed by EOCs citral, thymol, trans-cinnamaldehyde, linalool, α-pinene, carvacrol, (-)-terpinen-4-ol and allo-ocimene. A comparable proportion of the EOs tested (25.7%; n = 45) showed an activity larger than 0.5 against both *Candida* species (Fig. 2c). Of these, 30 were rich in at least one of the previously mentioned EOCs, while 7 of the 15 remaining EOs primarily contained components that were not included in our collection. By contrast, the iVMAA was zero for representatives of all antifungal classes commonly used in clinical practice (*i*.*e*. amphotericin B, caspofungin, fluconazole, terbinafine and 5-flucytosine) when tested at five to ten times their MIC (data not shown). This lack of iVMAA was to be expected, considering the relatively high molecular weight and concomitant low vapour pressure at room temperature and/or high solubility in water of the tested molecules. Surprisingly, ethanol also failed to inhibit growth when tested in the VMS assay using the standard set-up despite its known antimicrobial activity and relatively high vapour pressure at room temperature. However, since it is known that relatively high concentrations of ethanol are needed to inhibit microbial growth^29,30^, we theorised that higher volumes were needed to observe iVMAA. Indeed, when testing ethanol at ten times the standard volume, *i*.*e*. 4 × 200 µL, an iVMAA of 3.5 to 4 was obtained (data not shown).

### The major components present in an EO largely determine the presence or absence of iVMAA

**Figure 3:**
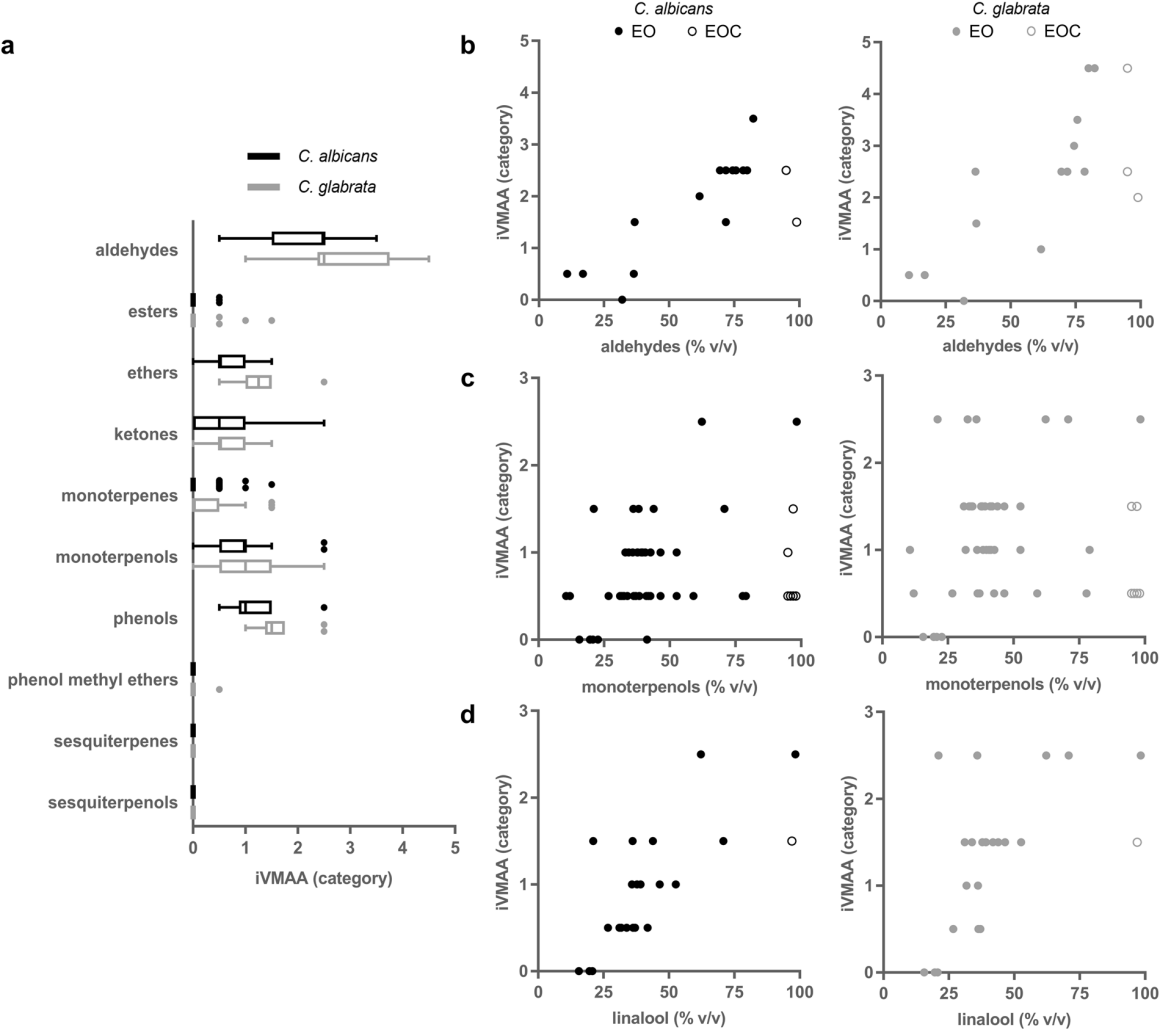
The major components present in an EO largely determine the presence or absence of iVMAA. (**a**) Tukey boxplots representing the iVMAA of EO(C)s (n = 209) categorised by the chemical class present at the highest concentration after combining all EOCs (>10%) belonging to the same chemical class. EOs for which one single major component could not be determined or for which this major component belonged to other chemical classes than defined in this paper were excluded (n = 3). (**b**) Correlations between the iVMAA of an EO(C) and its aldehyde concentration (>10%, n = 17) for *C*. *albicans* (top; ρ = 0.709, p = 0.0020) and for *C*. *glabrata* (bottom; ρ = 0.694, p = 0.0025). (**c**) Correlations between the iVMAA of an EO(C) and its monoterpenol concentration (>10%, n = 48) for *C*. *albicans* (top; ρ = 0.341, p = 0.018) and *C*. *glabrata* (bottom; ρ = 0.176, p = 0.23). (**d**) Correlations between the iVMAA of an EO(C) and its linalool concentration (>10%, n = 22) for *C*. *albicans* (top; ρ = 0.736, p < 0.0001) and *C*. *glabrata* (bottom; ρ = 0.6065, p = 0.0028).

To study the effect of the major individual EOCs in the EOs on their iVMAA, we categorised all EOs in 10 chemical classes. These classes were defined based on the number of carbon atoms and the presence of specific functional groups in the dominant EOCs (Fig. 3a and SI 1). Categorization occurred by the chemical class present at the highest concentration after combining all EOCs (>10% v/v) belonging to the same chemical class. This revealed that EO(C)s rich in aldehydes *e*.*g*. citronellal, citral and trans-cinnamaldehyde showed the highest iVMAA, followed by EO(C)s rich in phenols, *e*.*g*. carvacrol and thymol, monoterpenols, *e*.*g*. linalool and terpinen-4-ol, ethers, *e*.*g*. 1,8-cineol, and ketones such as carvone.

While monoterpenol-rich EO(C)s were among the most active classes, EO(C)s rich in sesquiterpenols *e*.*g*. farnesol did not show iVMAA. Furthermore, an absence of iVMAA was shown for EOs that were mainly rich in sesquiterpenes such as β-caryophyllene, and iVMAA was very limited in EOs that were rich in phenol methyl ethers, *e*.*g*. methyleugenol. The high iVMAA observed for aldehyde-rich EOs was not only a result of the presence of aldehyde(s) but was also strongly correlated with the quantity of aldehyde(s) present in the EO (Fig. 3b). In contrast, for monoterpenol-rich EOs and the corresponding EOCs, the correlation between iVMAA and monoterpenol concentration was weak for *C*. *albicans*, while no correlation could be demonstrated for *C*. *glabrata* (Fig. 3c). While there was a moderate to strong correlation between the concentration of the tertiary monoterpenol linalool in an EO and its iVMAA (Fig. 3d), we did not observe a correlation between the concentration of geraniol, a primary monoterpenol, in an EO and its iVMAA (SI 5). Together this shows that while major components in EOs often determine the presence or absence of iVMAA, they are not always responsible for the observed biological effects. It is thus advisable to also be attentive to the contributions of minor components when performing this kind of analysis.

### *C.glabrata* shows an average higher susceptibility to the iVMAA of EO(C)s than *C*. *albicans*

**Figure 4:**
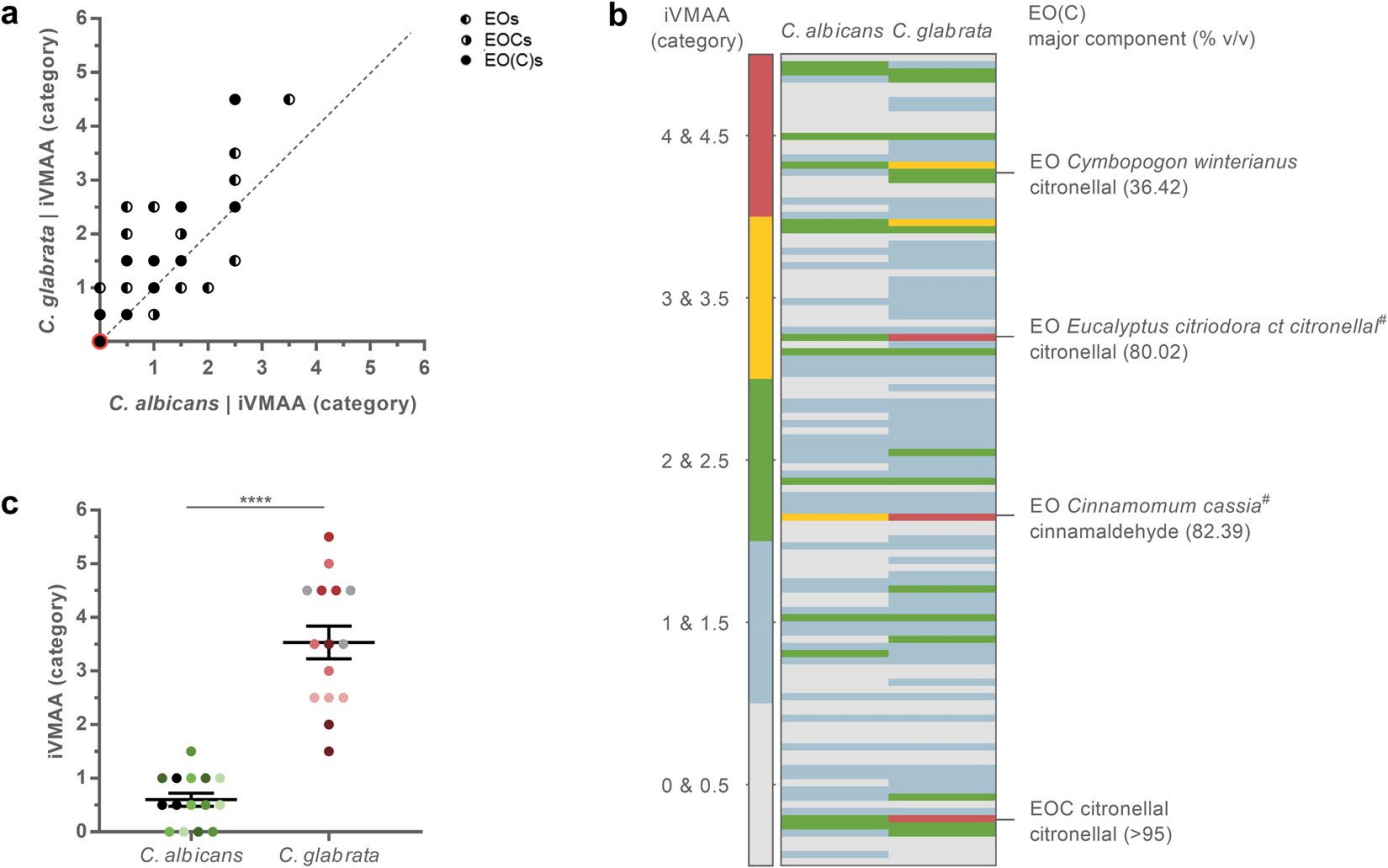
*C*. *glabrata* is on average more susceptible to the iVMAA of EO(C)s than *C*. *albicans*. (**a**) Scatterplot of the correlation (ρ = 0.930, p < 0.0001) between iVMAAs of EO(C)s (n = 212) against *Candida albicans and C*. *glabrata*. (**b**) Heat map of iVMAAs (>0 against at least one species) indicating the most differentially active of the EO(C)s (n = 113) against *C*. *albicans* and *C*. *glabrata*. ^#^ indicates that EO originates from an organic cultivar. (**c**) Graph showing iVMAA of citronellal in our VMS set-up against five *C*. *albicans* strains (SC5314 in black, and four clinical isolates) and five *C*. *glabrata* strains (ATCC2001 in grey, and four clinical isolates). Three independent experiments per strain are shown in the same colour and error bars represent standard error of the mean. iVMAA = inhibitory vapour-phase-mediated antimicrobial activity. ****p < 0.0001.

While the iVMAAs of the EO(C)s against the two *Candida* species tested correlated strongly (Fig. 4a), the overall susceptibility of the two species differed significantly (p < 0.0001). Despite the lower susceptibility of *C*. *glabrata* to antifungals in general^8,25,31^, it showed a higher average susceptibility to EO(C)s in the VMS assay compared to *C*. *albicans*. This higher susceptibility was evidenced by (i) more EO(C)s showing an iVMAA against *C*. *glabrata* (n = 113) than against *C*. *albicans* (n = 100) and (ii) on average a higher iVMAA of the EO(C)s (iVMAA > 0; n = 113) against *C*. *glabrata* ($\bar{x}$ = 1.28 with a 95% CI between 1.13 and 1.44) than against *C*. *albicans* ($\bar{x}$ = 0.916 with a 95% CI between 0.783 and 1.05). This resulted in more data points above the diagonal in the correlation analysis (Fig. 4a) and a clear contrast between the iVMAA of the two species visualised with a heat map (Fig. 4b). This indicates that EOs, and most likely specific EOCs within these EOs, can show a specific activity. To find those EO(C)s that showed the largest differential iVMAA against both species, the false discovery rate method was applied using a Q-value of 1%, *i*.*e*. accepting that 1% of the declared discoveries were false positives^32^. This resulted in four discovered EO(C)s *i*.*e*. organic EO *Eucalyptus citriodora* ct citronellal (t-ratio = 6.10; degrees of freedom (d.f.) = 452), EOC citronellal (t-ratio = 5.63; d.f. = 452), EO *Cymbopogon winterianus* (t-ratio = 4.22; d.f. = 452) and organic EO *Cinnamomum cassia* (t-ratio = 4.22; d.f. = 452) (Fig. 4b). Alternatively, when performing t-tests corrected for multiple comparisons (α = 0.05), the same EO(C)s were shown to have a differential iVMAA against both species. All EO(C)s found were aldehyde-rich *i*.*e*. the EOC citronellal, two EOs rich in citronellal and one EO rich in trans-cinnamaldehyde (Fig. 4b).

When a Q-value of 2% was applied, two additional EOs were shown to be differentially active including *Leptospermum petersonii* (t-ratio = 3.29; d.f. = 452), thereby finding all EO(C)s present in our collection that contain citronellal at more than 10% (SI 1). Together, this gives a very strong indication that the aldehyde citronellal is responsible for the higher iVMAA observed against *C*. *glabrata*. Although organic EO *Cinnamomum cassia* was also differentially active, its main component trans-cinnamaldehyde itself was not, and no other EOs rich in this EOC were found differentially active at the tested statistical cut-offs. Therefore, another component or a combination of components is most likely causing this differential activity. The other EO found with at a Q-value of 2% was *Ammi visnage* EO (t-ratio = 3.29; d.f. = 452), which contains linalool and two esters at more than 10% (SI 1).

The iVMAA of EOC citronellal was additionally determined against four clinical isolates of each species to exclude that the higher susceptibility to citronellal of *C*. *glabrata* compared to *C*. *albicans* was strain-dependent (Fig. 4c). An unpaired t-test with Welch’s correction (t = 8.90; d.f. = 18) corroborated that the species *C*. *glabrata* is more susceptible to EOC citronellal than *C*. *albicans*.

## Discussion

Over the past decade, there has been a renewed interest in investigating the biological activities and application potential of EO(C)s. Although data on the anti-*Candida* activity of EO(C)s have been summarised recently^33,34^, it is often impossible to make reliable comparisons between results obtained in different studies because of different methods used. Moreover, often only a few EO(C)s are included in each study. Together, this results in poor documentation of the antifungal activity of EO(C)s.

While the broth microdilution assay is considered the gold standard for the detection of antimicrobial activity in solution^35,36^, it fails to detect VMAA of volatiles. Moreover, unless it has been taken into account in the design of an experiment, VMAA may lead to false positive results^22,23^. Therefore, we developed the VMS assay which quantifies the activity of a volatile on microorganisms in liquid culture, whereas other vapour-phase assays quantify the antimicrobial activity of the vapour-phase itself ^37–41^. Hence, the antimicrobial activity measured in both classes of assays is intrinsically different. Volatiles present in the volatility centre cause the inhibitory and cidal activities observed in the VMS assay^23^. Here, we used HS-SPME-GC-MS to additionally prove the physical presence of these volatiles, using citral isomers as an example, in all wells of the microtiter plate at different timepoints (Fig. 1d). The quantity of both isomers of citral decreased when moving away from the volatility centre following a near negative exponential distribution. A similar trend was observed for the antifungal activity of the EO of *Litsea citrata* (Fig. 1c and SI 2), which is rich in both citral isomers (71.80%). This concurs with what would be expected *i*.*e*. the strongest antifungal activity near the centre of volatility which gradually decreases when moving to the edges of the microtiter plate. However, this does not imply that citral was solely responsible for the observed effect in the wells as an EO typically consists of many minor components which all can contribute to the antifungal activity. To further characterise and as such allow predictions of the behaviour of volatiles in the VMS assay, it would be interesting to study the behaviour of a large array of EO(C)s in the VMS assay by measuring the concentrations of volatiles in the corresponding wells. This can be done, for example by analysing the headspace of the wells using HS-SPME combined with chromatographic analysis. By determining the *in vitro* growth-inhibitory activity of a large EO(C) collection against well-characterised strains of two pathogenic *Candida* species using the VMS and broth microdilution assay, we generated reliable and directly comparable data on the antimicrobial activity of EO(C)s (Fig. 2a). We found no correlation between iVMAA and MIC (Fig. 2b), which demonstrates that the iVMAA is a valuable additional measure to obtain a more complete picture of the antimicrobial potential of a volatile by incorporating volatility and antimicrobial activity. The dissimilarity between MIC and iVMAA was further illustrated by the very low iVMAA shown by EOs rich in phenol methyl ethers and phenol methyl ether EOCs (Fig. 3a), whereas some phenol methyl ethers tested here show potent anti-*Candida* activity in the broth microdilution assay^23^. While most of the EO(C)s that we studied showed VMAA, none of the representatives of each class of antifungals currently used in clinical practice^42^ showed iVMAA, indicating that this characteristic differentiates EO(C)s from currently used classes of antifungals. Furthermore, the high correlation between the iVMAA of EO(C)s against the two *Candida* species (Fig. 4a), shows that both microorganisms are overall similarly susceptible to EO(C)s. This might reflect a non-specific mode of action, such as membrane destabilisation, often ascribed to EO(C)s^14,33^. However, *C*. *glabrata* showed on average a higher susceptibility to EO(C)s compared to *C*. *albicans*. While multiple EO(C)s were shown to differentially affect both *Candida* species (Fig. 4b), the EOC citronellal was the strongest differential activity (Fig. 4b,c). In case the molecular target of citronellal is common in both *Candida* species, it would thus be more sensitive in *C*. *glabrata*. The activity of citronellal against *Candida* species has been shown before, and the proposed mode of action is an alteration of membrane homeostasis associated with decreased ergosterol content^43^. On the other hand, the higher susceptibility of *C*. *glabrata* might indicate a more specific mode of action. The fact that we could not show differential activity for the related aldehyde citral, which structurally only differs from citronellal by one additional double bond between positions 2 and 3 and is known for its strong antimicrobial activity^44,45^, points in the direction of such a specific mode of action.

The VMS assay is very versatile and is only limited by the architecture of standard 96-well microtiter plates. Obvious assay modifications would be (i) altering the size of the volatility-centre, which would only imply redefining the categories (*e*.*g*. for a volatility-centre of one well in SI 6a), and (ii) testing multiple volatiles in the same microtiter plate (*e*.*g*. with two volatility-centres of four wells). The latter would, for instance, allow for the detection of synergies between different antimicrobials acting via their vapour-phase (SI 6b). Most of our experiments were performed with two EO(C)s on the same plate and the maximum possible iVMAA that could be measured thus corresponded to category 5.5. The highest iVMAA observed in our experimental set-up was category 4.5, exhibited by the organic EO of *Cinnamomum cassia* of which the anti-*Candida* activity in liquid cultures is well-documented^46–48^. Moreover, (iii) multiple volumes of the same volatile could be tested in parallel to determine the minimum volume necessary to inhibit cell growth in all the wells of category one (*e*.*g*. with four one-well volatility-centres distributed over the microtiter plate in SI 6c). By (iv) using standard multi-well plates with a higher number of wells, the resolution could be increased allowing the detection of more subtle differences, and thus a more detailed characterisation of the VMAA. The VMS assay was tested extensively using pathogenic fungi. However, it can be used (v) to assess the influence of any volatile on anything that fits into the wells of a microtiter plate, and that can be assayed in such a format^25,49–51^.

A basic classification of the EO(C)s in our collection revealed that the presence/absence of iVMAA could often be predicted from the chemical class that predominates in the EOs, with aldehyde-, phenol-, monoterpenol-, ether- and ketone-rich EOs showing the highest iVMAA. This was substantiated by a high correlation between the aldehyde content or the monoterpenol linalool content of EOs, and the iVMAA exhibited by these EOs. Conversely, iVMAA was absent in EOs rich in sesquiterpenols and sesquiterpenes (Fig. 3a). This can partially be explained by the weak anti-*Candida* activity of terpenes in general. However, iVMAA not only reflects antimicrobial activity but depends greatly on volatility and the aqueous solubility of the bioactive compound(s), which is highly influenced by *e*.*g*. temperature and duration of the experiment. For example, it is known that phenol methyl ethers, sesquiterpenes and -terpenols show a much lower evaporation than monoterpenes and -terpenoids at 35 °C^52^.

In conclusion, this study illustrates the potent anti-*Candida* activity of EO(C)s by testing an extended collection of common EOs and EOCs. We demonstrate anti-*Candida* activity of EO(C)s both in liquid cultures and via their vapour-phase, as assessed in the VMS assay. Thanks to the procedural similarities between the VMS assay and the broth microdilution assays, the former can become a standard assay for the detection of VMA and can complement the latter. Thus, the detection of VMA allows a more complete description of the antimicrobial activity of molecules, thereby boosting the search for new antimicrobials and expanding the application potential of existing ones.

## Materials and Methods

### Essential oils (EOs), essential oil components (EOCs), antifungals and ethanol

All EOs (n = 175; SI 1) and all highly enriched EOCs (n = 37; SI 4) were purchased from and certified by Pranarôm International S.A. and Sigma-Aldrich, respectively. Chemical analysis of the EOs was performed by GC-FID using the NF ISO 11024-1/2 standard (list of components present at >10% per EO in SI 1). All EOs under study are dissimilar *i*.*e*. originating from different plant parts and/or from (non-)organic cultivation. EOs were considered rich in a component if that component was present at more than 10%. The only EOC that was solid at room temperature was dissolved in dimethyl sulfoxide (DMSO; proportion Thymol:DMSO 5:1; Sigma-Aldrich) (see table SI 4) and pure DMSO was used as a negative control. All EO(C)s were aliquoted in brown sterile glass vials (Screening Devices), coded to blind the experiments and stored at 4 °C. The antifungals caspofungin, flucanozole, terbinafine and 5-flucytosine purchased from and certified by Sigma-Aldrich were dissolved (5 mg/mL) in DMSO, DMSO, methanol (Acros Organics) and milli-Q water (Merck Millepore), respectively, and stored at −20 °C. The antifungal amphotericin B (Gibco by life technologies) was dissolved in fungizone (Gibco life technologies) at a concentration of 250 µg/mL prior to storage at −20 °C. The antiseptic ethanol (Fisher chemical) was stored according to supplier’s instructions.

### *Candidaalbicans* and *Candidaglabrata*

*C*. *albicans* strain SC5314^53^, *C*. *glabrata* strain ATCC 2001 and four randomly selected clinical isolates from blood of each species were used in this study. All strains were maintained on YPD agar plates (10 g/L yeast extract, 20 g/L bactopeptone, 20 g/L glucose, 15 g/L Difco™ agar) and refreshed weekly. Prior to experiments, the strains were grown overnight at 35 °C on 47 g/L Sabouraud agar plates.

### Preparation of the cell inocula

The cell density of a small loop of overnight propagated cells, suspended in 1x phosphate-buffered saline (PBS; 8 g/L sodium chloride, 0.2 g/L potassium chloride, 1.44 g/L disodium hydrogen phosphate, 0.24 g/L potassium dihydrogen phosphate) was estimated by measuring the optical density at 600 nm (OD_600_). For the illustration of the growth inhibitory VMAA of EO *Litsea citrata* (Fig. 1c and SI 1), YPD medium (10 g/L yeast extract, 20 g/L bactopeptone, 20 g/L glucose) was used. For all other experiments, the cell suspension measured at OD_600_, was made in Roswell Park Memorial Institute-1640 (RPMI) medium (Sigma-Aldrich), and prepared in accordance with CLSI guidelines^25^. Briefly, the medium was buffered with 3-(N-morpholino) propanesulfonic acid (MOPS; Sigma-Aldrich) and filter-sterilised over a 0.20 µm non-pyrogenic Nalgene^TM^ filter (Fisher Scientific).

### Vapour-phase-mediated susceptibility assay (VMS assay)

The VMS assay was based on the protocol for the vapour‐phase‐mediated patch assay described before^23^. In the standard VMS set-up, 200 µL of a 5 × 10^3^ cells/mL inoculum was added to all wells of a polystyrene 96-well microtiter plate with U-shaped wells (Greiner Bio-One), except for wells H1 and H12 which served as blanks and contained 200 µL medium. Next, 20 µL of the EO(C) under study was added on top of the cell suspension in wells D/E6-7. Alternatively, when testing the complete EO(C) collection, two EO(C)s were tested per microtiter plate. In such cases, the first EO(C) was added on top of the cell suspension in wells D/E3-4, while the second EO(C) was added on top of the cell suspension in wells D/E9-10. For each run, one microtiter plate without EO(C)s was included as an external negative control and one microtiter plate with 2 µL of EOC trans-cinnamaldehyde was added to wells D/E3-4 as a positive control. The microtiter plates were covered with a lid and statically incubated for 24 hours at 35 °C while limiting air draughts. The OD_600_ was measured with a multi-well plate reader (Synergy H1, BioTek Germany) after resuspending the cells. Wells in which growth was visually absent (OD_600_ ≤ 0.07) or wells with OD_600_ < 10% of OD_600_ of the external control plate after correcting for the blank were counted, excluding wells to which the EO(C) was added and blanks, to determine iVMAA and iVMAA_90_, respectively. The resulting number of wells was categorized according to the categories defined in Fig. 1b. Only wells located in columns 1–3 and columns 10–12 were included and multiplied by two before categorisation, to exclude possible interactions between two EO(C)s tested in the same plate. All EO(C)s with an iVMAA larger than zero against at least one of the two *Candida* species were tested three times.

### Headspace solid‐phase micro-extraction gas chromatography mass spectrometry (HS-SPME-GC-MS)

For the sample preparation, the VMS assay was run for one and 24 hours with 200 µL double-distilled water instead of medium in the wells of the microtiter plate. The content of wells belonging to the same category was pooled and mixed, after which 700 µL was transferred to 10 mL headspace crimp vials (Restek). For SPME sample extraction, a Supelco StableFlex Fiber with 50/30 µm DVB/Carboxen/PDMS-coated fibre coating (100 μm, Supelco Inc., 57284-U) was used, mounted in a TriPlus RSH autosampler (Thermo Fischer Scientific). The fibre was placed in the headspace of each sample for 10 min, positioned in the agitator and heated to 70 °C. Sample desorption occurred in the SSL injection port of the GC at 250 °C for 3 min. Before and after sampling, the fibre was inserted in an SPME fibre conditioning station for 5 min for thermal desorption at 250 °C. GC-MS analysis was performed using a Trace 1300 Gas Chromatograph (Thermo Fischer Scientific) with a Stabilwax capillary column, 60 m × 0.25 mm i.d. × 0.25 μm f.t. (Restek), coupled to an ISQ QD Single Quadrupole Mass Spectrometer (Thermo Fischer Scientific). Carrier gas: helium 2 mL/min; split ratio: 15; temperature: 35 °C for 2 min, then raised from 35 to 215 °C at 10 °C/min and a 2 min hold at 215 °C; transfer line temperature: 250 °C; detector temperature: 175 °C; mass range: m/z 40−300. For both enantiomers α-citral and β-citral, response signals were obtained by integration of the peaks in the extracted Ion channel (*m/z* 69) at retention times 16.98 min and 16.40 min, respectively. The identity of the components was confirmed by comparison of the apex MS spectra to the NIST Mass Spectral Search Program, version 2.0 f. As the obtained signal is proportional to the concentration in the sample as was determined by linear calibration after sampling of a dilution series (α-citral: R² = 99.220 and β-citral: R² = 99.536), the relative concentrations can be compared by comparing peak areas.

### Broth microdilution assay

The broth microdilution assay was performed in accordance with the CLSI protocol^25^ with few adaptations. Briefly, 100 µL of a 1 × 10^4^ cells/mL inoculum was added to all wells of a polystyrene 96-well microtiter plate with U-shaped wells, with the exception of wells serving as blanks to which 200 µL RPMI-MOPS was added. One part of each EO(C) was dissolved in eight parts (v/v) DMSO, after which a two-fold dilution series ranging from 0.25% (v/v) EO(C) to 0.0078% (v/v) EO(C) was prepared in RPMI-MOPS. One hundred µL of each dilution was added to the respective wells with cell suspension, resulting in a final cell concentration of 5 × 10^3^ cells/mL and final EO(C) concentrations ranging from 0.13% (v/v) to 0.0039% (v/v). Plates were sealed with an acetate Corning^®^ Cap Mat and incubated for 24 hours at 35 °C. The MIC was determined as the lowest EO(C) concentration required for visual growth inhibition.

### Statistical analyses

Statistical analyses were performed using GraphPad Prism version 7.03 and included all biological repeats. Figures show category-corrected averages (in accordance with Fig. 1a,b) of the biological repeats. A mono-exponential equation (one-phase decay equation), which resulted in the best goodness of fit (adjusted R²), was used to describe the distribution of EOC citral across the microtiter plate. For correlation analyses, the Spearman rank correlation coefficient (ρ) was calculated. The population-wide susceptibility of both *Candida* species to EO(C)s was compared using the Wilcoxon matched-pairs signed rank test. Differentially active EO(C)s were identified using multiple t-tests performed on all EO(C)s with an iVMAA larger than zero against at least one of the species, followed by two methods for post-hoc testing *i*.*e*. the false discovery rate (FDR) and the statistical significance approach^32,54^. The citronellal susceptibility of five *C*. *albicans* and five *C*. *glabrata* strains was compared using the unpaired t-test with Welch’s correction.

### Data availability

The datasets generated and analysed during the current study are not publicly available due to possible valorisation of the data but are available from the corresponding author on reasonable request.

## Electronic supplementary material

Supplementary data
