# Supplementary material for: Essential oils and their components are a class of antifungals with potent vapour-phase-mediated anti-Candida activity
Source: Sci Rep. 2018 Mar 2;8:3958. doi: 10.1038/s41598-018-22395-6 (PMC5834617; doi:10.1038/s41598-018-22395-6)
Supplement: Supplementary file 1 — Supplementary data [file 41598_2018_22395_MOESM1_ESM.pdf]

Essential oils and their components are a class of antifungals with potent vapour-phase-mediated anti-*Candida* activity

short title: Vapour-phase-mediated antimicrobial activity

Adam F. Feyaerts[a,b,1], Lotte Mathé[a,b,1], Walter Luyten[c], Stijn De Graeve[a,b], Katrien Van Dyck[a,b], Lize Broekx[a,b] and Patrick Van Dijck[a,b,2]

Supplementary data:

SI1: EOs used in this study with their major components (≥10%) and assigned chemical class of components. AP = aerial parts; AP-S = aerial parts - seeds; B = bark; BB = berry branches; F = fruits; FB = flower buds; FL = flowers; FT = flowering tops; H = herbs; L = leaves; LT = leafy twigs; N = needles; O = oleoresin; P = peels; R = roots; RH = rhizome; T = twigs; T + B = twigs + bark; TF = twigs flowers; W = wood. # = organic EO; ct = chemotype; ssp = subspecies; cv = cultivar; var = variety. When no EOCs present at > 10% (n=2): only EOC at highest is concentration shown.

Plant species	Part of plant	Lot number	Major components (≥ 10% v/v)	% (v/v)	Chemical class
Abies alba	N	OF11341	α-PINENE	34.30	monoterpenes
			LIMONENE	19.42	monoterpenes
			β-PINENE	17.71	monoterpenes
			CAMPHENE	12.90	monoterpenes
Abies balsamea[#]	N	OF11276	β-PINENE	33.96	monoterpenes
			δ3-CARENE	14.67	monoterpenes
			α-PINENE	12.80	monoterpenes
Abies sibirica	N	OF10586	BORNYL ACETATE	28.45	esters
			CAMPHENE	22.00	monoterpenes
			δ3-CARENE	12.91	monoterpenes
			α-PINENE + α-THUJENE	11.91	monoterpenes + monoterpenes
Achillea millefolium	FT	OF10749	SABINENE	17.78	monoterpenes
			GERMACRENE D	16.36	sesquiterpenes
			β-PINENE	14.92	monoterpenes
Ammi visnaga[#]	S	OF8569	LINALOOL	35.82	monoterpenols
			ISOAMYL 2-METHYLBUTYRATE	15.53	esters
			AMYL ISOBUTYRATE	10.80	esters
Anethum graveolens	F	OF8420	CARVONE	50.44	ketones
			LIMONENE	43.08	monoterpenes
Angelica archangelica	R	OF1127	α-PINENE	22.01	monoterpenes
			δ3-CARENE	16.42	monoterpenes
			α-PHELLANDRENE	11.32	monoterpenes
			β-PHELLANDRENE	10.76	monoterpenes
Apium graveolens	F	OF10289	LIMONENE	63.69	monoterpenes
			β-SELINENE	17.55	sesquiterpenes
Artemisia dracunculus	FT	OF10105	ESTRAGOLE	79.36	phenol methyl ethers

Artemisia herba alba	FT	OF11480	α-THUJONE	61.45	ketones
			CAMPHOR	12.47	ketones
			β-THUJONE	10.39	ketones
Cananga odorata extra	FL	OF10390	GERMACRENE D	14.61	sesquiterpenes
Cananga odorata totum#	FL	OF9867	GERMACRENE D	18.23	sesquiterpenes
			b-CARYOPHYLLENE	12.45	sesquiterpenes
Canarium luzonicum	O	OF9870	LIMONENE	51.76	monoterpenes
			α-PHELLANDRENE	12.87	monoterpenes
Carum carvi	F	000027	CARVONE	54.09	ketones
			LIMONENE	43.78	monoterpenes
Cedrelopsis grevei#	W	OF9859	ISHWARANE	21.35	sesquiterpenes
Cedrus atlantica	W	OF10992	β-HIMACHALENE	43.34	sesquiterpenes
			α-HIMACHALENE	16.75	sesquiterpenes
Cedrus atlantica#	W	OF10799	β-HIMACHALENE	42.39	sesquiterpenes
			α-HIMACHALENE	17.09	sesquiterpenes
			γ-HIMACHALENE	10.17	sesquiterpenes
Cedrus deodara	W	OF10214	β-HIMACHALENE	38.29	sesquiterpenes
			α-HIMACHALENE	16.76	sesquiterpenes
			γ-HIMACHALENE	10.16	sesquiterpenes
Chamaemelum nobile	FL	OF10863	ISOBUTYL ANGELATE + ISOAMYL METHACRYLATE	32.93	esters + esters
			ISOAMYL ANGELATE	17.40	esters
Chamaemelum nobile#	FL	OF11255	METHYLAMYL ANGELATE	17.16	esters
			METHALLYL ANGELATE	12.84	esters
Cinnamomum camphora ct cineole#	L	OF11065	1,8-CINEOLE	56.28	ethers
			SABINENE	13.19	monoterpenes
Cinnamomum camphora ct linalool	W	OF10369	LINALOOL	98.35	monoterpenols
Cinnamomum cassia	T	OF10584	E-CINNAMALDEHYDE	78.45	aldehydes
			trans-o-METHOXY-CINNAMALDEHYDE	10.75	phenol methyl ethers
Cinnamomum cassia#	T	OF10588	E-CINNAMALDEHYDE	82.39	aldehydes
Cinnamomum zeylanicum	B	OF10850	E-CINNAMALDEHYDE	61.69	aldehydes

Cinnamomum zeylanicum	L	OF9780	EUGENOL	74.35	phenol methyl ethers
Cinnamosma fragrans#	L	OF10651	1,8-CINEOLE	42.28	ethers
Cistus ladaniferus ct pinene#	T	OF10502	CAMPHENE	29.98	monoterpenes
			α-PINENE	15.47	monoterpenes
			BORNYL ACETATE	11.79	esters
Citrus aurantifolia	F	OF10400	LIMONENE	44.61	monoterpenes
			γ-TERPINENE + Trans-β-OCIMENE	12.67	monoterpenes + monoterpenes
Citrus aurantium ssp amara	L	OF10467	LINALYL ACETATE	52.94	esters
			LINALOOL	20.71	monoterpenols
Citrus aurantium ssp amara	P	OF10404	LIMONENE	93.37	monoterpenes
Citrus aurantium ssp amara#	L	OF11484	LINALYL ACETATE	46.65	esters
			LINALOOL	26.70	monoterpenols
Citrus aurantium ssp amara#	FL	OF10993	LINALOOL	46.47	monoterpenols
Citrus aurantium ssp bergamia	P	OF10862	LIMONENE	47.94	monoterpenes
			LINALYL ACETATE	23.60	esters
Citrus bergamia ssp bergamia#	P	OF11052	LIMONENE	46.80	monoterpenes
			LINALYL ACETATE	26.33	esters
Citrus limon	P	OF11188	LIMONENE	67.01	monoterpenes
			β-PINENE	11.85	monoterpenes
Citrus limon	L	OF3114	LIMONENE	41.19	monoterpenes
			β-PINENE	18.38	monoterpenes
			CITRAL	10.80	aldehydes
Citrus limon#	P	OF11178	LIMONENE	64.31	monoterpenes
			β-PINENE	13.88	monoterpenes
Citrus paradisii	P	OF9436	LIMONENE	93.79	monoterpenes
Citrus paradisii#	P	OF9722	LIMONENE	94.61	monoterpenes
Citrus reticulata	L	OF9239	METHYL N-METHYLANTHRANILATE	54.32	esters
			γ-TERPINENE	23.83	monoterpenes
Citrus reticulata	P	OF3457	LIMONENE	87.50	monoterpenes
Citrus reticulata#	P	OF10644	LIMONENE	70.74	monoterpenes

			γ-TERPINENE	18.31	monoterpenes
Citrus sinensis	P	OF9238	LIMONENE	95.10	monoterpenes
Citrus sinensis#	P	OF11321	LIMONENE	95.09	monoterpenes
Copaifera officinalis	O	OF10996	β-CARYOPHYLLENE	54.20	sesquiterpenes
Coriandrum sativum	F	OF11183	LINALOOL	70.83	monoterpenols
Corydothymus capitatus	FT	OF11481	CARVACROL	66.65	phenols
Crithmum maritimum#	FT	OF9373	γ-TERPINENE + Trans-β-OCIMENE	48.83	monoterpenes + monoterpenes
			β-PHELLANDRENE	19.70	monoterpenes
Cuminum cyminum	F	OF9607	CUMINAL	31.94	aldehydes
			γ-TERPINENE	18.32	monoterpenes
			β-PINENE	16.71	monoterpenes
			p-CYMENE	16.31	monoterpenes
Cupressus sempervirens var stricta	T	OF10218	α-PINENE + α-THUJENE	58.01	monoterpenes + monoterpenes
			δ3-CARENE	13.18	monoterpenes
Cupressus sempervirens var stricta#	T	OF10846	α-PINENE + α-THUJENE	49.83	monoterpenes + monoterpenes
			δ3-CARENE	15.19	monoterpenes
Curcuma longa#	R	OF10876	ar-TURMERONE	38.76	ketones
			ar-TURMERONE	23.37	ketones
Cymbopogon citratus	AP	OF10881	CITRAL	69.57	aldehydes
Cymbopogon flexuosus	H	OF9994	CITRAL	74.40	aldehydes
Cymbopogon giganteus#	L	OF9770	trans-p-1,7-MENTHA-8,9-DIEN-2-OL	19.60	monoterpenols
			cis-p-MENTHA-2,8-DIEN-1-OL	17.84	monoterpenols
			cis-p-1,7-MENTHA-8,9-DIEN-2-OL	15.20	monoterpenols
			LIMONENE	12.56	monoterpenes
Cymbopogon martinii var motia	AP	OF10011	GERANIOL	78.99	monoterpenols
Cymbopogon martinii var motia#	AP	OF9950	GERANIOL	77.78	monoterpenols
			GERANYL ACETATE	10.96	esters
Cymbopogon nardus	AP	OF2106	GERANIOL	22.63	monoterpenols
Cymbopogon winterianus	AP	OF10851	CITRONELLAL	36.42	aldehydes

			GERANIOL	20.25	monoterpenols
			CITRONELLOL	12.19	monoterpenols
Daucus carota var sativus	F	OF11585	CAROTOL	38.56	sesquiterpenols
Daucus carota var sativus[#]	AP	OF4113	α-PINENE	23.79	monoterpenes
			β-BISABOLENE + β-SELINENE	21.59	sesquiterpenes + sesquiterpenes
			trans-METHYLISOEUGENOL	16.93	phenol methyl ethers
Elettaria cardamomum	F	OF12267	TERPENYL ACETATE	35.60	esters
			1,8-CINEOLE	32.76	ethers
Eucalyptus citriodora ct citronellal[#]	L	OF10647	CITRONELLAL	80.02	aldehydes
Eucalyptus dives ct piperitone[#]	L	OF10872	PIPERITONE + BICYCLOGERMACRENE	39.04	ketones + sesquiterpenes
			α-PHELLANDRENE	22.20	monoterpenes
			1,8-CINEOLE + β -PHELLANDRENE	10.79	ethers + monoterpenes
Eucalyptus globulus	L	OF10646	1,8-CINEOLE	80.51	ethers
Eucalyptus globulus[#]	L	OF11274	1,8-CINEOLE	81.99	ethers
Eucalyptus polybractea ct cryptone[#]	L	OF11142	p-CYMENE	28.14	monoterpenes
			SPATHULENOL	10.98	sesquiterpenol
Eucalyptus radiata ssp radiata	L	OF10865	1,8-CINEOLE	71.80	ethers
Eucalyptus radiata ssp radiata[#]	L	OF10720	1,8-CINEOLE	71.93	ethers
*Eucalyptus smithii**	L	OF9370	1,8-CINEOLE	77.31	ethers
Eugenia caryophyllus	FB	OF9948	EUGENOL	83.33	phenol methyl ethers
			EUGENYL ACETATE	11.58	esters
Eugenia caryophyllus[#]	FB	OF10583	EUGENOL	81.78	phenol methyl ethers
			EUGENYL ACETATE	12.90	esters
Ferula gummosa	O	OF12273	β-PINENE	56.53	monoterpenes
			δ3-CARENE	11.97	monoterpenes
Foeniculum vulgare[#]	AP- S	OF10796	trans-ANETHOLE	85.18	phenol methyl ethers
Fokiena hodginsii	BB	OF11589	FOKIENOL	39.08	sesquiterpenols
			NEROLIDOL	31.65	sesquiterpenols
Gaultheria fragrantissima[#]	L	OF11051	METHYL SALYCATE	99.39	esters
Gaultheria procumbens	L	OF11068	METHYL SALYCATE	99.37	esters

Helichrysum italicum ssp serotinum	FT	OF9441	NERYL ACETATE	19.88	esters
			γ-CURCUMENE + γ-MUUROLENE	15.04	sesquiterpenes + sesquiterpenes
			α-PINENE	14.77	monoterpenes
Helichrysum italicum ssp serotinum[#]	FT	OF10622	NERYL ACETATE	20.25	esters
			α-PINENE	15.47	monoterpenes
			γ-CURCUMENE	11.55	sesquiterpenes
Hyssopus officinalis var officinalis	FT	OF11287	ISOPINOCAMPHONE	36.22	ketones
			PINOCAMPHONE	18.68	ketones
			β-PINENE	10.74	monoterpenes
Illicium verum[#]	F	OF10399	trans-ANETHOLE	89.22	phenol methyl ethers
Inula graveolens[#]	FT	OF11143	BORNYL ACETATE	43.47	esters
			BORNEOL	19.59	monoterpenols
Juniperus communis ssp communis[#]	T + B	OF10654	α-PINENE + α-THUJENE	40.12	monoterpenes + monoterpenes
Juniperus communis var alpina[#]	T	OF4120	LIMONENE	26.29	monoterpenes
			β-PHELLANDRENE	16.71	monoterpenes
Juniperus oxycedrus	T	OF9732	δ-CADINENE	25.01	sesquiterpenes
			CUBENOL	11.33	sesquiterpenols
Juniperus virginiana	W	OF9364	THUJOPSENE	27.41	sesquiterpenes
			α-CEDRENE	24.11	sesquiterpenes
			CEDROL	16.77	sesquiterpenols
Laurus nobilis	L	OF11039	1,8-CINEOLE	41.40	ethers
Lavandula angustifolia spp angustifolia	FT	OF10864	LINALOOL	37.79	monoterpenols
			LINALYL ACETATE	33.41	esters
Lavandula angustifolia spp angustifolia[#]	FT	OF10951	LINALOOL	41.87	monoterpenols
			LINALYL ACETATE	31.34	esters
Lavandula latifolia	FT	OF10007	LINALOOL	39.10	monoterpenols
			1,8-CINEOLE + β-PHELLANDRENE	26.97	ethers + monoterpenes
			CAMPHOR	12.54	ketones
Lavandula latifolia[#]	FT	OF9693	LINALOOL	43.88	monoterpenols
			1,8-CINEOLE + β-PHELLANDRENE	25.92	ethers + monoterpenes

			CAMPHOR	10.82	ketones
Lavandula stoechas[#]	FT	OF11591	CAMPHOR	29.48	ketones
			FENCHONE	24.41	ketones
Lavandula x burnatii clone abrialis[#]	FT	OF10059	LINALOOL	36.12	monoterpenols
			LINALYL ACETATE	19.34	esters
Lavandula x burnatii clone grosso	FT	OF1743	LINALYL ACETATE	33.11	esters
			LINALOOL	31.07	monoterpenols
Lavandula x burnatii clone grosso[#]	FT	OF6689	LINALOOL	37.06	monoterpenols
			LINALYL ACETATE	29.68	esters
Lavandula x burnatii clone reydovan	FT	OF9108	LINALOOL	52.58	monoterpenols
			LINALYL ACETATE	18.43	esters
Lavandula x burnatii clone super	FT	OF10869	LINALOOL	36.28	monoterpenols
			LINALYL ACETATE	33.98	esters
Lavandula x burnatii clone super[#]	FT	OF10745	LINALOOL	33.86	monoterpenols
			LINALYL ACETATE	33.44	esters
Ledum groenlandicum[#]	TF	OF9694	SABINENE	25.18	monoterpenes
Leptospermum petersonii	L	OF10212	CITRAL	56.97	aldehydes
			CITRONELLAL	18.72	aldehydes
Levisticum officinale	R	OF9237	LIGUSTILIDE	42.85	phtalides
			(Z)-BUTYLIDENE PHTHALIDE	41.96	phtalides
Lippia citriodora	L	OF9771	LIMONENE	20.92	monoterpenes
			CITRAL + PIPERITONE	16.91	aldehydes + ketones
Litsea citrata	F	OF10225	CITRAL	71.80	aldehydes
			LIMONENE	12.90	monoterpenes
Litsea citrata[#]	F	OF11261	CITRAL	71.80	aldehydes
			LIMONENE	11.05	monoterpenes
Matricaria recutita	FL	OF10999	trans-β-FARNESENE	43.60	sesquiterpenes
Melaleuca alternifolia	L	OF11248	TERPINENE-4-OL	41.35	monoterpenols
			γ-TERPINENE	20.64	monoterpenes
Melaleuca alternifolia[#]	L	OF10388	TERPINENE-4-OL	38.38	monoterpenols

			γ-TERPINENE	22.23	monoterpenes
			α-TERPINENE	11.07	monoterpenes
Melaleuca cajeputi	L	OF10662	1,8-CINEOLE	69.15	ethers
Melaleuca cajeputi#	L	OF11405	1,8-CINEOLE	61.09	ethers
			α-TERPINEOL + γ-MUUROLENE	11.13	monoterpenols + sesquiterpenes
Melaleuca quinquenervia ct cineol	L	OF10731	1,8-CINEOLE	57.07	ethers
Melaleuca quinquenervia ct cineol#	L	OF10956	1,8-CINEOLE	55.18	ethers
Melissa officinalis	AP	OF10300	β-CARYOPHYLLENE	23.46	sesquiterpenes
			CITRAL	36.76	aldehydes
			GERMACRENE D	11.52	sesquiterpenes
Mentha arvensis	AP	OF10883	MENTHOL	42.68	monoterpenols
			MENTHONE	18.42	ketones
Mentha arvensis#	AP	OF9728	MENTHOL	39.61	monoterpenols
			MENTHONE	13.59	ketones
Mentha citrata	AP	OF9449	LINALYL ACETATE	36.47	esters
			LINALOOL	31.71	monoterpenols
Mentha x piperita	AP	OF10867	MENTHOL	37.61	monoterpenols
			MENTHONE	23.98	ketones
Mentha x piperita#	AP	OF11594	MENTHOL	33.05	monoterpenols
			MENTHONE	23.80	ketones
Mentha pulegium	AP	OF9582	PULEGONE	84.14	ketones
Mentha spicata#	AP	OF12639	CARVONE	56.35	ketones
			LIMONENE	12.68	monoterpenes
			trans-CARVEOL	11.9	monoterpenols
Myristica fragrans	F	OF10590	α-PINENE + α-THUJENE	23.79	monoterpenes + monoterpenes
			SABINENE	15.01	monoterpenes
			β-PINENE	14.25	monoterpenes
Myroxylon balsamum var pereiras	O	OF1706	DMSO		
Myrtus communis ct cineole#	L	OF9864	α-PINENE	51.52	monoterpenes
			1,8-CINEOLE	23.87	ethers

			LIMONENE	10.36	monoterpenes
Myrtus communis ct myrtenyl acetate	L	OF9391	1,8-CINEOLE	29.37	ethers
			α-PINENE + α-THUJENE	22.40	monoterpenes + monoterpenes
			MYRTENYL ACETATE	18.59	esters
			LIMONENE	11.78	monoterpenes
Myrtus communis ct myrtenyl acetate[#]	L	OF10882	1,8-CINEOLE	30.33	ethers
			α-PINENE	22.81	monoterpenes
			MYRTENYL ACETATE	18.18	esters
			LIMONENE	11.63	monoterpenes
Nardostachys jatamansi[#]	R	OF11047	CALARENE	9.49	sesquiterpenes
Ocimum basilicum ssp basilicum[#]	FT	OF10861	ESTRAGOLE	71.57	phenol methyl ethers
			LINALOOL	19.59	monoterpenols
Ocimum sanctum	L	OF11260	EUGENOL	50.45	phenol methyl ethers
			β-CARYOPHYLLENE	22.47	sesquiterpenes
Origanum compactum	FT	OF10299	CARVACROL	43.67	phenols
			THYMOL	19.52	phenols
			γ-TERPINENE + Trans-β-OCIMENE	16.16	monoterpenes + monoterpenes
Origanum compactum[#]	FT	OF11283	CARVACROL	39.44	phenols
			THYMOL	23.17	phenols
			γ-TERPINENE + Trans-β-OCIMENE	13.70	monoterpenes + monoterpenes
			p-CYMENE	10.89	monoterpenes
Origanum heracleoticum	FT	OF11288	CARVACROL	66.96	phenols
Origanum majorana	FT	OF10217	TERPINEOL	22.90	monoterpenols
			γ-TERPINENE	14.85	monoterpenes
			cis-THUJANOL	11.39	monoterpenols
Origanum majorana[#]	FT	OF9776	TERPINEOL	23.01	monoterpenols
			cis-THUJANOL	15.11	monoterpenols
			γ-TERPINENE	13.77	monoterpenes
Origanum majorana ct thujanol	FT	OF10871	cis-THUJANOL	21.41	monoterpenols
			TERPINEOL	19.67	monoterpenols

			γ-TERPINENE	12.46	monoterpenes
Pelargonium x asperum cv Bourbon[#]	L	OF12053	CITRONELLOL + d-CADINENE	22.20	monoterpenols + sesquiterpenes
			GERANIOL + CALAMENENE	16.14	monoterpenols + sesquiterpenes
			CITRONELLYL FORMATE	10.96	esters
Pelargonium x asperum	L	OF9858	CITRONELLOL	32.40	monoterpenols
			GERANIOL	14.07	monoterpenols
Petroselinum crispum	L	OF11587	1,3,8-p-MENTHATRIENE	20.75	monoterpenes
			α-PINENE	18.76	monoterpenes
			MYRISTICINE	13.64	phenol methyl ethers
			β-PINENE	12.74	monoterpenes
Picea mariana[#]	N	OF10298	BORNYL ACETATE	28.58	esters
			CAMPHENE	19.09	monoterpenes
			α-PINENE	14.24	monoterpenes
Pimenta racemosa	F	OF9871	EUGENOL	46.32	phenol methyl ethers
			β-MYRCENE	25.87	monoterpenes
			CHAVICOL	10.35	phenols
Pinus pinaster	O	OF11277	α-PINENE + α-THUJENE	74.27	monoterpenes + monoterpenes
			β-PINENE	17.07	monoterpenes
Pinus ponderosa	N	OF11050	β-PINENE	38.12	monoterpenes
			ESTRAGOLE	18.91	phenol methyl ethers
			δ3-CARENE	17.47	monoterpenes
Pinus sylvestris	N	OF11339	α-PINENE	40.96	monoterpenes
			β-PINENE	20.22	monoterpenes
			δ3-CARENE	16.12	monoterpenes
Pinus sylvestris[#]	N	OF2115	α-PINENE	40.76	monoterpenes
			β-PINENE	24.62	monoterpenes
Piper nigrum	F	OF9540	β-CARYOPHYLLENE	20.83	sesquiterpenes
			LIMONENE	19.07	monoterpenes
			α-PINENE + α-THUJENE	15.23	monoterpenes + monoterpenes
			β-PINENE	13.10	monoterpenes

			δ3-CARENE	12.94	monoterpenes
Pistacia lentiscus#	LT	OF9359	β-MYRCENE	16.73	monoterpenes
			α-PINENE	16.58	monoterpenes
			LIMONENE	13.96	monoterpenes
Pogostemon cablin	FT	OF10211	PATCHOULOL	30.15	sesquiterpenols
			α-BULNESENE	18.00	sesquiterpenes
			α-GUAIENE	14.98	sesquiterpenes
Pogostemon cablin#	FT	OF9954	PATCHOULOL	28.99	sesquiterpenols
			α-BULNESENE	19.70	sesquiterpenes
			α-GUAIENE	15.47	sesquiterpenes
Ravensara aromatica	B	OF9044	ESTRAGOLE	90.01	phenol methyl ethers
Ravensara aromatica#	L	OF11431	LIMONENE	16.45	monoterpenes
Rosmarinus officinalis ct camphor#	FT	OF11044	α-PINENE	21.00	monoterpenes
			1,8-CINEOLE	19.05	ethers
			CAMPHOR	17.28	ketones
Rosmarinus officinalis ct cineole	FT	OF10655	1,8-CINEOLE	42.01	ethers
			α-PINENE	13.72	monoterpenes
			CAMPHOR	12.85	ketones
Rosmarinus officinalis ct cineole#	FT	OF10408	1,8-CINEOLE	44.38	ethers
			α-PINENE	10.76	monoterpenes
			CAMPHOR	10.07	ketones
Rosmarinus officinalis ct verbenone#	FT	OF10075	α-PINENE + α-THUJENE	42.70	monoterpenes + monoterpenes
Salvia lavandulifolia	FT	OF11046	CAMPHOR	29.99	ketones
			1,8-CINEOLE	26.41	ethers
Salvia officinalis	FT	OF10880	α-THUJONE	37.64	ketones
			β-THUJONE	12.74	ketones
Salvia officinalis#	FT	OF9241	α-THUJONE	24.73	ketones
			CAMPHOR	22.23	ketones
			1.8-CINEOLE	11.19	ethers
Salvia sclarea#	FT	OF9454	LINALYL ACETATE	71.98	esters

			LINALOOL	15.56	monoterpenols
Santalum austrocaledonicum var austrocaledonicum	W	OF11042	(Z)-α-SANTALOL	43.93	sesquiterpenols
			(Z)- β-SANTALOL	18.51	sesquiterpenols
Satureja hortensis	FT	OF3340	γ-TERPINENE	42.45	monoterpenes
			CARVACROL	29.95	phenols
			p-CYMENE	10.07	monoterpenes
Satureja montana	FT	OF11247	CARVACROL	43.51	phenols
			γ-TERPINENE + Trans-β-OCIMENE	16.62	monoterpenes + monoterpenes
			p-CYMENE	13.86	monoterpenes
Solidago canadensis[#]	AP	OF10723	GERMACRENE D	28.46	sesquiterpenes
			α-PINENE	12.87	monoterpenes
Tanacetum annuum	L	OF10287	SABINENE	16.87	monoterpenes
			CAMPHOR	12.87	ketones
Thuya occidentalis	T	OF9451	α-THUJONE	52.15	ketones
			FENCHONE	16.59	ketones
Thymus mastichina	FT	OF9049	1.8-CINEOLE	52.44	ethers
			LINALOOL	20.98	monoterpenols
Thymus satureioides	FT	OF10106	BORNEOL	28.01	monoterpenols
			α-TERPINEOL	12.64	monoterpenols
Thymus satureioides[#]	FT	OF10589	BORNEOL	28.36	monoterpenols
			α-TERPINEOL	14.25	monoterpenols
			CAMPHENE	10.73	monoterpenes
Thymus serpyllum	FT	OF10659	p-CYMENE	15.62	monoterpenes
			CARVACROL	14.69	phenols
			THYMOL	12.93	phenols
			GERANIOL	10.51	monoterpenols
Thymus vulgaris ct geraniol	FT	OF9453	GERANIOL	59.00	monoterpenols
			GERANYL ACETATE	15.73	esters
Thymus vulgaris ct linalool[#]	FT	OF12998	LINALOOL	62.19	monoterpenols
Thymus vulgaris ct thymol[#]	FT	OF10842	THYMOL	36.39	phenols

			p-CYMENE	22.71	monoterpenes
			γ-TERPINENE	12.77	monoterpenes
Thymus zygis	FT	OF9050	THYMOL	48.13	phenols
			p-CYMENE	21.22	monoterpenes
Trachyspermum ammi	F	OF9576	THYMOL	35.24	phenols
			γ-TERPINENE	34.65	monoterpenes
			p-CYMENE	22.41	monoterpenes
Tsuga canadensis#	N	OF10884	BORNYL ACETATE	31.43	esters
			α-PINENE	20.69	monoterpenes
			CAMPHENE	15.3	monoterpenes
Valeriana officinalis	R	OF9234	BORNYL ACETATE	35.57	esters
			CAMPHENE	23.79	monoterpenes
Vetiveria zizanoïdes	R	OF10233	β-VETIVENENE	8.32	sesquiterpenes
Zingiber officinalis#	RH	OF11621	α-ZINGIBERENE	24.00	sesquiterpenes
			β-SESQUIPHELLANDRENE	10.85	sesquiterpenes
			CAMPHENE	10.34	monoterpenes

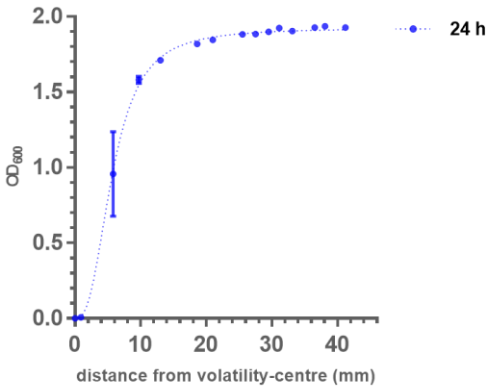

SI 2: The growth inhibitory effect of a volatile is largest in wells close to the volatility-centre. Spectrophotometric assessment of *C. albicans* growth inhibition by *Litsea citrata* EO in the VMS assay at 24 hours. One data point represents the average of four to 12 wells, in accordance with number of wells per category shown in figure 1b. Error bars represent standard deviation.

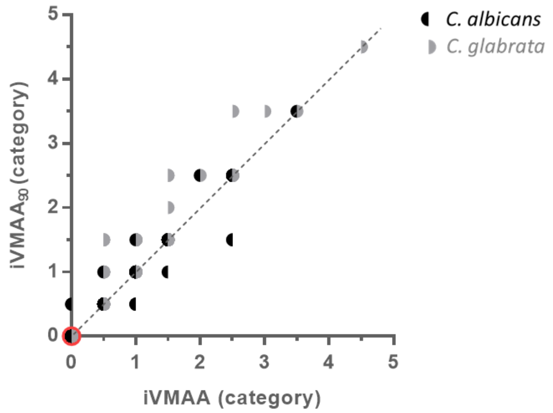

SI 3: There is a very strong correlation between iVMAA and iVMAA$_{90}$ for each *Candida* species. Scatterplot showing the correlation between iVMAA and iVMAA$_{90}$ of EO(C)s (n=212) against *C. albicans* (ρ=0.991, p<0.0001) and *C. glabrata* (ρ=0.991, p<0.0001). Red symbol indicates negative control DMSO. iVMAA = inhibitory vapour-phase-mediated antimicrobial activity (visual assessment). iVMAA$_{90}$ = iVMAA resulting in 90% reduction of growth as compared to control growth (spectrophotometric assessment).

SI4: Highly enriched EOCs used in this study with their purity and assigned chemical class.

EOC	minimum purity (%)	chemical class
trans-ANETHOLE	99	phenol methyl ethers
BENZYL BENZOATE	99	esters
(-)-BORNYL ACETATE	95	esters
CARVACROL	99	phenols
(-)-CARVONE	98	ketones
(+)-CARVONE	98	ketones
β-CARYOPHYLLENE	80	sesquiterpenes
1,8-CINEOLE	99	ethers
trans-CINNAMALDEHYDE	99	aldehydes
CITRAL	95	aldehydes
CITRONELLAL	95	aldehydes
CITRONELLOL	95	monoterpenols
p-CYMENE	97	monoterpenes
ESTRAGOLE	98	phenol methyl ethers
EUGENOL	99	phenol methyl ethers
FARNESOL	95	sesquiterpenols
FARNESYL ACETATE	NA	esters
GERANIOL	98	monoterpenols
GERANYL ACETATE	97	esters
(+)-LIMONENE	97	monoterpenes
(-)-LIMONENE	95	monoterpenes
LINALOOL	97	monoterpenols
LINALYL ACETATE	97	esters
METHYL EUGENOL	98	phenol methyl ethers
MYRCENE	90	monoterpenes
NEROL	97	monoterpenols
ALLO-OCIMENE	80	monoterpenes
β-OCIMENE	90	monoterpenes
α-PHELLANDRENE	NA	monoterpenes
α-PINENE	98	monoterpenes
(-)-β-PINENE	99	monoterpenes
(+)-PULEGONE	90	ketones
(-)-TERPINEN-4-OL	95	monoterpenols
γ-TERPINENE	97	monoterpenes
α-TERPINEOL	96	monoterpenols
TERPINYL ACETATE	95	esters
THYMOL (5:1 DMSO)	99	phenols

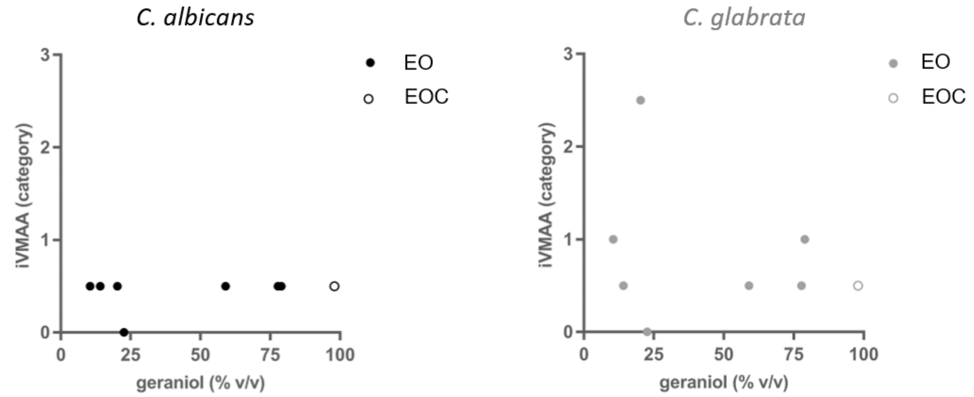

SI 5: No correlation could be observed between the geraniol concentration of an EO(C) and its iVMAA against each *Candida* species. Correlations between the iVMAA of an EO(C) and its geraniol concentration (>10%,n=8) for *C. albicans* (ρ=0.0825, p>0.99) and *C. glabrata* (ρ=-0.217, p=0.62).

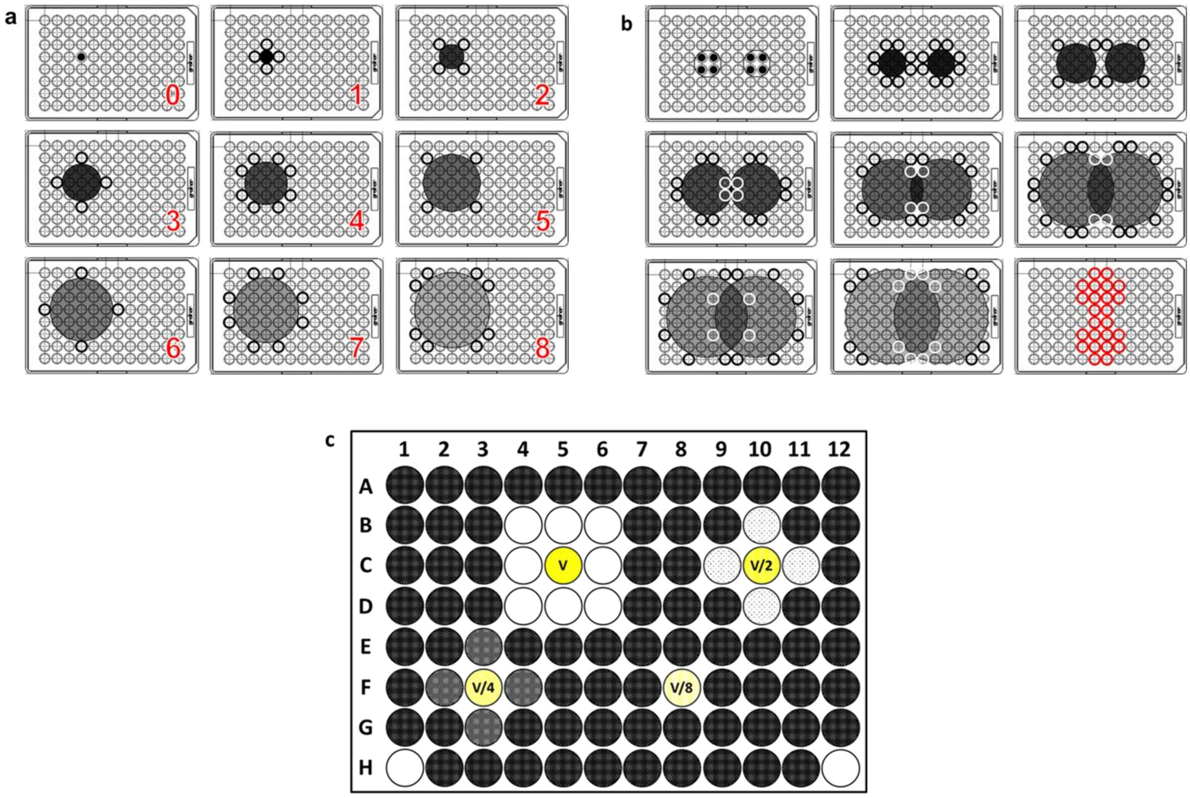

SI 6: The VMS assay is versatile as demonstrated by these examples of alternative set-ups. a: Changing the size of the volatility centre affects the categories. Upper-left: volatility-centre of one well. Upper-middle to bottom-right: First eight categories associated with a volatility-centre of size one well. **b:** Using two volatility-centres allows for the detection of synergies between two volatiles. Upper-left: two volatility-centres; Upper-middle to bottom-middle: The first seven stages associated with using two volatility-centres of four wells; Bottom-right: The interaction zone between the two volatiles added in their respective volatility-centre in which synergies can be detected. **c:** Testing multiple volumes of the same volatile using volatily-centres of one well allows for the determination of the minimal volume necessary to inhibit cell growth in all wells of the first category. V = volume.
